# Molecular mechanism of amyloidogenic mutations in hypervariable regions of antibody light chains

**DOI:** 10.1016/j.jbc.2021.100334

**Published:** 2021-01-26

**Authors:** Georg J. Rottenaicher, Benedikt Weber, Florian Rührnößl, Pamina Kazman, Ramona M. Absmeier, Manuel Hitzenberger, Martin Zacharias, Johannes Buchner

**Affiliations:** 1 Center for Integrated Protein Science Munich at the Department Chemie, Technische Universität München, Garching, Germany; 2 Center for Integrated Protein Science Munich at the Physik-Department, Technische Universität München, Garching, Germany

**Keywords:** AL amyloidosis, antibody light chain, amyloid fibrils, protein folding, protein aggregation

## Abstract

Systemic light chain (AL) amyloidosis is a fatal protein misfolding disease in which excessive secretion, misfolding, and subsequent aggregation of free antibody light chains eventually lead to deposition of amyloid plaques in various organs. Patient-specific mutations in the antibody V_L_ domain are closely linked to the disease, but the molecular mechanisms by which certain mutations induce misfolding and amyloid aggregation of antibody domains are still poorly understood. Here, we compare a patient V_L_ domain with its nonamyloidogenic germline counterpart and show that, out of the five mutations present, two of them strongly destabilize the protein and induce amyloid fibril formation. Surprisingly, the decisive, disease-causing mutations are located in the highly variable complementarity determining regions (CDRs) but exhibit a strong impact on the dynamics of conserved core regions of the patient V_L_ domain. This effect seems to be based on a deviation from the canonical CDR structures of CDR2 and CDR3 induced by the substitutions. The amyloid-driving mutations are not necessarily involved in propagating fibril formation by providing specific side chain interactions within the fibril structure. Rather, they destabilize the V_L_ domain in a specific way, increasing the dynamics of framework regions, which can then change their conformation to form the fibril core. These findings reveal unexpected influences of CDR-framework interactions on antibody architecture, stability, and amyloid propensity.

Amyloidoses comprise a family of protein misfolding diseases in which disease-specific precursor proteins aggregate into highly ordered amyloid fibrils (1). These fibrils form amyloid plaques, which are deposited either systemically or in an organ-specific manner causing severe damage (2). The most common systemic disease in this context is amyloid light chain (AL) amyloidosis, in which an antibody light chain (LC) acts as the precursor protein that eventually forms amyloid fibers (3, 4). In healthy individuals, plasma cells secrete IgG antibodies, which consist of two LCs and two heavy chains covalently linked by disulfide bridges. Each of the LCs is made up of an N-terminal variable (V_L_) domain and a C-terminal constant (C_L_) domain (5). In AL amyloidosis, malignant monoclonal plasma cells overproduce and secrete LCs into the blood stream leading to very high concentrations of circulating LCs (6, 7). The malignant plasma cells often emerge in the course of an underlying plasma cell dyscrasia (*e.g.*, multiple myeloma) (7). During the complex maturation process responsible for the creation of antibody binding diversity, these LCs acquire amyloidogenic point mutations mostly in the V_L_ domain, which is the main constituent of fibrils in AL amyloidosis (8, 9, 10). These point mutations and (in many cases) proteolytic cleavage of the LC to produce the free V_L_ domain are key factors for disease onset and progression (11, 12, 13, 14). Yet, the exact mechanism by which certain mutations favor amyloid formation of antibody domains remains largely elusive. Recent cryo-EM and solid-state NMR studies of AL amyloid fibrils show that the fold of the fibril core is strikingly different from the native Ig fold suggesting that a complete structural rearrangement has to occur in the process of fibril formation (15, 16, 17, 18, 19, 20). For a large number of AL cases, it has been shown that the decrease in thermodynamic stability of the V_L_ domain is a decisive factor for amyloidogenicity (21, 22, 23, 24, 25). However, also nondestabilizing mutations in the V_L_ domain can induce fibril formation, thus other factors such as LC dimerization, structural changes, and conformational dynamics also need to be taken into account (26, 27, 28, 29, 30).

A major enigma in this context is how different mutations shift V_L_ domains toward the fibrillary pathway, especially if these mutations are not in the conserved framework but in the variable antigen binding regions called complementarity determining regions (CDRs). These are solvent-exposed loops connecting β-strands (31). In order to recognize a large variety of antigens, CDRs need to tolerate a high sequence variability, which in turn suggests that CDR point mutations should not strongly affect the thermodynamic stability and aggregation propensity of the antibody domain (32, 33, 34, 35). In 2017, however, Annamalai *et al.* (36) reported the crystal structure and the fibril morphology of a V_L_ domain (FOR005-PT) obtained from an AL amyloidosis patient with mainly cardiac involvement in which four out of the five mutations are located in the CDRs (according to the Kabat/Chothia domain numbering). Since current models cannot explain the amyloidogenic character of this variant, we set out to determine which of these mutations drive amyloid aggregation and found that specifically two of the CDR mutations are causative for fibril formation. Our findings further show that seemingly minor side chain alterations, even in poorly conserved CDRs, can destabilize the entire V_L_ domain and drive it toward misfolding and amyloid aggregation.

## Results

### Sequence and structure analysis

**Figure 1:**
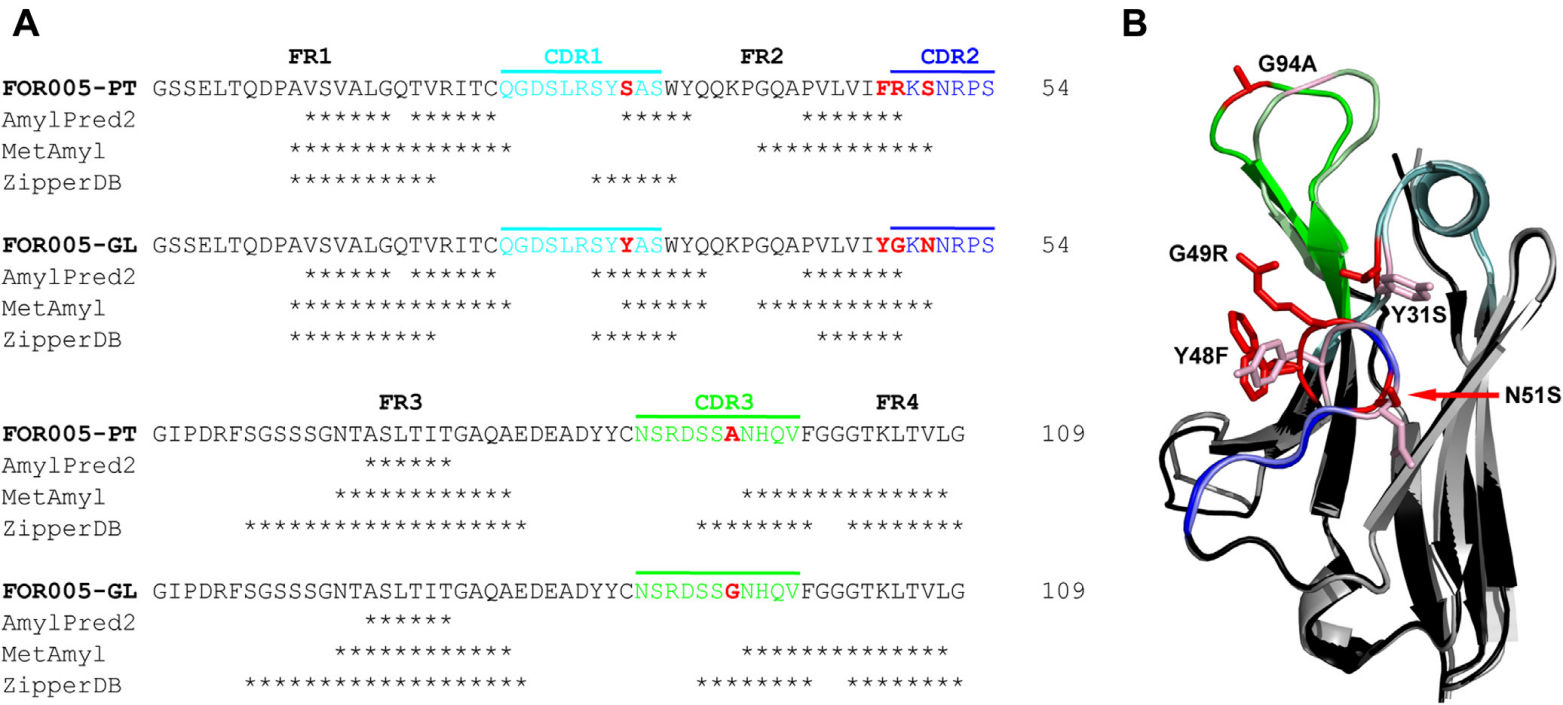
**Sequence and structural analyses of FOR005-PT and FOR005-GL.***A*, sequence alignment of patient and germline V_L_ shows the five point mutations highlighted in *red* and the variable CDR loops in *cyan* (CDR1), *blue* (CDR2), and *green* (CDR3). Predictions of amyloidogenic regions by three different tools overlap well indicating that the point mutations do not introduce new amyloid driving segments. Aggregation-prone positions are indicated by *asteriks*. The sequence numbering as derived from Annamalai *et al*. starts with the first serine residue, Ser1. The N-terminal glycine in our sequence results from using NcoI during subcloning of the FOR005 gene constructs and is, therefore, numbered as Gly0. *B*, structural alignment of FOR005-PT shown in *black* (PDB: 5L6Q) with the homology model derived for FOR005-GL depicted in *gray*. The homology model was created using the SWISS-MODEL server and the template structure 5BV7. CDRs are colored according to the sequence alignment in *A*. Mutated positions are shown in *red* on the patient V_L_ and *light red* on the germline V_L_ domain with side chains depicted as *sticks*. For F48 on the patient structure two rotamers are shown. The structural comparison suggests rearrangements of loop conformations in CDR2 and CDR3.

In 2017, Annamalai *et al.* (36) reported the cDNA sequence and crystal structure (PDB: 5L6Q) of an amyloid forming V_L_ domain (FOR005-PT) derived from a patient with cardiac LC amyloidosis. We used IgBLAST, IMGT, and abYsis to determine the corresponding germline sequence (FOR005-GL) with the highest possible protein sequence identity for this amyloidogenic V_L_ (37, 38, 39, 40). FOR005-PT belongs to the λ3l LC subfamily (gene segments: *IGLV3-19/IGLJ2*). The related germline λ3r has been reported to be associated with AL amyloidosis (41, 42). Five point mutations were identified in the patient-derived V_L_ domain (Y31S, Y48F, G49R, N51S, G94A) compared with the germline sequence (Fig. 1*A*), but it was not clear which mutation causes amyloid aggregation. Four of them are located in the hypervariable CDRs according to the Kabat and Chothia numbering systems (43, 44). The mutation Y31S is located in the CDR1 region, Y48F lies in the conserved framework 2 region (FR2) right next to the beginning of CDR2. The CDR2 comprises a short, protruding loop segment containing the mutations G49R and N51S. The fifth mutation, G94A, is located in the hypervariable CDR3 loop. Residue conservation analysis with Consurf revealed the lowest degree of conservation (Consurf score = 1) for all four CDR mutations and an average conservation degree (Consurf score = 5) for the framework residue F48 in the patient sequence (Fig. S1) (45). We further assessed aggregation-prone regions and individual mutational effects by applying the prediction tools AmylPred2, MetAmyl, and ZipperDB (46, 47, 48). Amyloidogenicity predictions by these tools did not suggest significant alterations of the amyloid aggregation or steric zipper propensity of the patient V_L_ sequence in comparison with its corresponding germline sequence (Fig. 1*A*). To explain structural effects of mutations in more detail, we created a homology model of FOR005-GL based on the template structure 5BV7, which exhibits 98.2% sequence identity, using the SWISS-MODEL web server (49). Structural alignments of the homology model with the crystal structure of FOR005-PT (PDB: 5L6Q) showed that the overall structure was conserved, although the conformations of the CDR2 and CDR3 loops are altered (Fig. 1*B*). However, one needs to take into account that homology models are merely an approximation of the actual native protein structure.

### FOR005-PT and FOR005-GL differ substantially in fibril formation propensity and thermodynamic stability

To test the biophysical properties of the proteins directly, we produced patient and germline V_L_ domains recombinantly in *E.coli* and purified them to homogeneity. The far-UV circular dichroism (CD) spectra of the purified proteins showed that both are properly folded and possess the typical β-sheet-rich immunoglobulin fold as indicated by the minimum at around 218 nm in the far-UV region (Fig. S2) (50). Near-UV CD spectra, which represent a specific tertiary structure fingerprint, were highly similar for the two proteins. Thus, FOR005-PT and FOR005-GL seem to have nearly identical tertiary structure and topology. Additionally, analytical ultracentrifugation (AUC) was performed to assess the quaternary structure. As indicated by sedimentation coefficients of 1.52 and 1.59 S, respectively, both the patient and germline V_L_ domains are monomeric in solution (Fig. S2).

**Figure 2:**
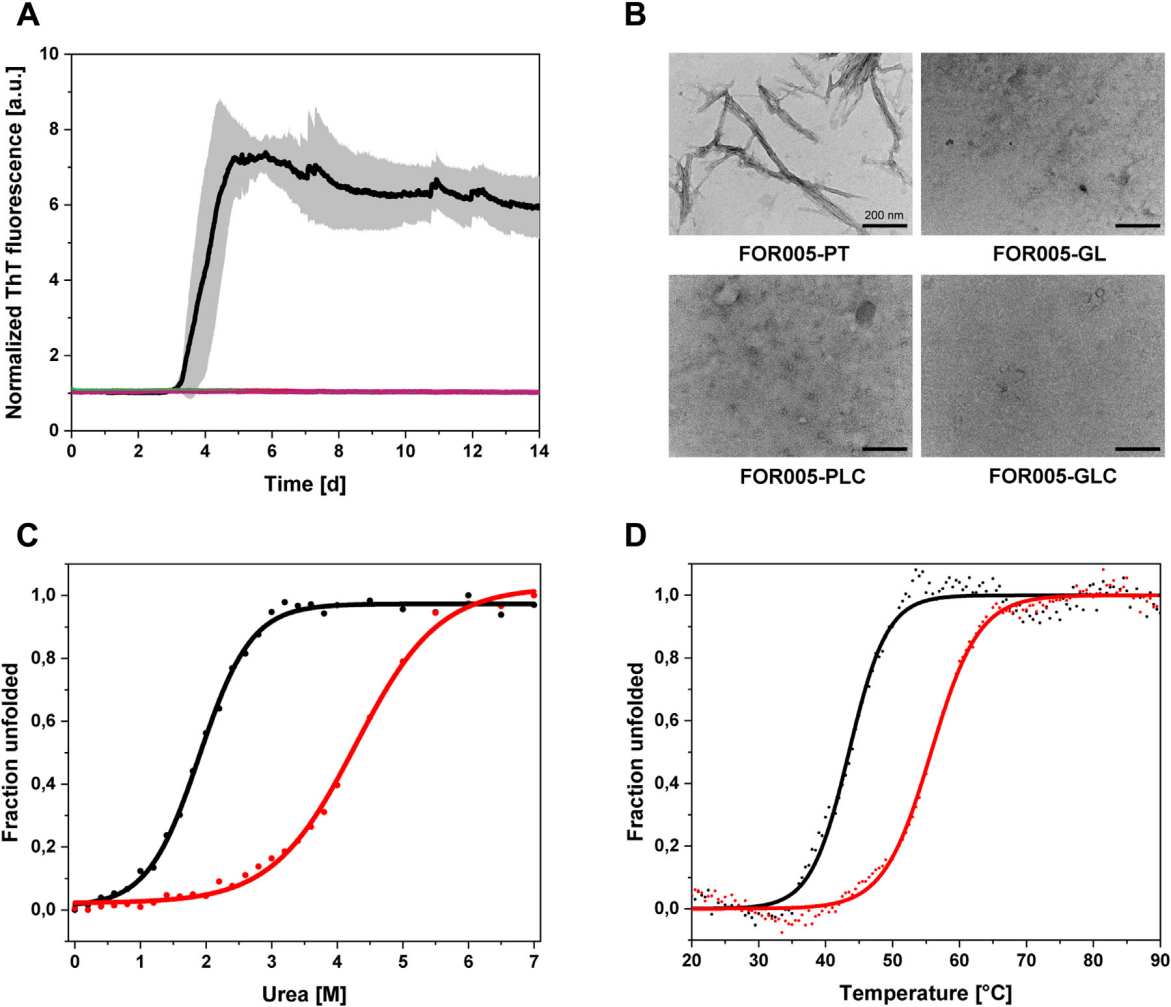
**Fibril formation propensity of the patient V**_**L**_**domain.***A*, thioflavin T-binding kinetics of FOR005-PT (*black*), -GL (*red*), -PLC (*light green*), and -GLC (*light purple*) obtained at 37 °C and pH 7.4 under continuous shaking. The increase in fluorescence shows that the patient V_L_ domain is the only protein engaging in amyloid fibril formation after approximately 3 days. Connecting the patient V_L_ domain with the C_L_ domain to form a full-length LC completely inhibited fibril formation. All kinetic curves were normalized to a fluorescence start value of 1. *B*, TEM micrographs of samples from finished ThT assays were recorded after negative stain with uranyl acetate. The amyloid fibers of FOR005-PT can be seen in the *upper left panel*, the scale bar represents 200 nm. *C*, for chemical unfolding transitions, 1 μM protein was equilibrated with increasing concentrations of urea over night at room temperature. Fluorescence spectra (λ_ex_ = 280 nm/λ_em_ = 300–400 nm) were recorded at 25 °C in a 96-well plate. The transition of FOR005-PT is shown with *black dots*, the data for FOR005-GL is shown as *red dots*. The *black* and *red sigmoidal lines* represent the individual fit functions. *D*, thermal unfolding transitions of FOR005-PT and -GL were obtained by recording CD signal at 205 nm while applying a temperature gradient from 20 to 90 °C with a heating rate of 1 °C/min. Sample concentration was 10 μM in PBS and the measurement was performed in a 1 mm quartz cuvette.

To test whether the two V_L_ domains differ in their fibril formation propensities, we incubated the proteins in phosphate buffered saline (PBS) at pH 7.4 and 37 °C under continuous shaking and monitored fibril formation *via* the thiazol-based fluorescent dye Thioflavin T (ThT), which specifically binds to the characteristic cross-β motif in amyloid fibrils (51). ThT-binding kinetics showed that the patient V_L_ domain starts to form amyloid fibrils *in vitro* after approximately 3 days, whereas the corresponding germline protein does not engage in amyloid aggregation (Fig. 2*A*, Table 1). To obtain direct evidence for the presence of fibrils in the samples, we performed transmission electron microscopy (TEM). The TEM micrographs showed fibrils only in the patient V_L_ sample and not in the germline control (Fig. 2*B*). Thus, the patient V_L_ behaved as expected and the germline protein does not show amyloidogenic behavior. FOR005 fibrils isolated from patient tissue contained only the V_L_ domain (35). Since the role of proteolytic cleavage of precursor LCs in amyloidosis is still only poorly understood (14), we purified full-length LCs of the patient and germline variants (FOR005-PLC and FOR005-GLC, respectively) to determine whether the patient LC is also amyloidogenic. We performed fibril formation assays and transition electron microscopy and found that both LCs did not engage in the amyloidogenic pathway (Fig. 2).

**Table 1:** Stability parameters, unfolding cooperativity, and fibril formation midpoints of FOR005 constructs.

V_L_ domain	T_m_	C_m_	m	t_50_ pH 7.4	t_50_ pH 6.4
	°C	M Urea	kJ mol^−1^ M^−1^	d	d
FOR005-PT	43.5 ± 0.14	1.90 ± 0.02	5.99 ± 0.33	3.8	2.8
FOR005-GL	56.3 ± 0.11	4.28 ± 0.04	4.33 ± 0.31	-	-
GL Y31S	56.1 ± 0.12	4.20 ± 0.03	5.12 ± 0.47	-	-
GL Y48F	55.4 ± 0.13	4.15 ± 0.03	4.56 ± 0.32	-	-
GL G49R	52.2 ± 0.10	2.91 ± 0.01	5.55 ± 0.13	-	-
GL N51S	54.4 ± 0.10	3.44 ± 0.02	5.01 ± 0.27	-	-
GL G94A	50.5 ± 0.17	3.25 ± 0.05	4.95 ± 0.99	-	-
GL Y31S/G94A	50.3 ± 0.15	3.31 ± 0.03	5.17 ± 0.58	-	-
GL Y48F/G94A	51.9 ± 0.10	3.22 ± 0.02	5.32 ± 0.28	-	-
GL G49R/G94A	47.0 ± 0.09	2.09 ± 0.02	6.69 ± 0.41	8.9	4.4
GL N51S/G94A	48.6 ± 0.08	2.56 ± 0.02	5.98 ± 0.33	-	11.5

Thermal transitions were obtained by recording the CD signal at 205 nm between 20 to 90 °C at a heating rate of 1 °C/min. Chemical unfolding transitions were obtained by fluorescence spectroscopy using 1 μM of each V_L_ domain with increasing concentrations of urea. Since both thermal and chemical unfoldings are irreversible, the stability parameters T_m_ and C_m_ represent apparent values. Transition midpoints and standard deviations were derived from a Boltzmann fit. Chemical unfolding data was also subjected to a two-state unfolding fit model to determine cooperativity and $\Delta G_{un}^{app}\;$ values (Table S1). Fibril formation assays were carried out at 37 °C, pH 7.4 or 6.4 under continouos shaking in a Tecan Genios platereader. The t_50_ values represent the time point at which fibril formation is 50% completed.

To determine differences between the two V_L_ domains regarding their thermodynamic properties in more detail, we investigated their stabilities by chemical and thermal denaturation experiments. Unfolding transitions in the presence of increasing urea concentrations were performed to assess the chemical domain stability and unfolding cooperativity of the V_L_ domains (Fig. 2, Table 1). The patient V_L_ domain showed a midpoint of unfolding at a urea concentration (C_m_) of 1.90 M, whereas for the germline domain, the midpoint is at 4.28 M urea (Fig. 2, Table 1). We assessed the reversibility of urea-induced unfolding by fluorescence spectroscopy and found that both V_L_ domains cannot be completely refolded into their native structure within 24 h at room temperature (Fig. S2). It should be noted, however, that the germline V_L_ exhibits a higher degree of unfolding reversibility than the patient variant (Fig. S2). We applied a two-state fit model to our transition data to calculate unfolding free energies (ΔG_un_). However, this is in principle only possible if unfolding is completely reversible. Since this is not the case under the conditions used (Fig. S2), these results do not represent true ΔG_un_ values but are rather apparent unfolding free energies ($\Delta G_{un}^{app}$) (Table S1).

In thermal denaturation experiments, the patient-derived protein exhibited a melting temperature (T_m_) of 43.5 °C, whereas the germline protein showed a melting temperature of 56.3 °C. The T_m_ values correspond to the temperatures at which 50% of the protein is unfolded. Since thermal unfolding of both FOR005-PT and FOR005-GL is also irreversible (Fig. S2), the obtained transition midpoints represent apparent melting temperatures (Table 1). These data show that the patient V_L_ domain has a significantly decreased thermodynamic stability compared with its germline counterpart (Table 1).

### Mutations in hypervariable regions affect domain stability and aggregation

While the results described above show that the patient-specific mutations affect conformational stability and fibril formation, it was not possible to rationalize which of the mutations are responsible for amyloidogenesis. To determine the specific effects of each of the five point mutations, we replaced them individually in the germline sequence by the respective patient residues (Y31S, Y48F, G49R, N51S, and G94A). CD spectroscopy and AUC analysis of the mutants showed that all point mutants adopted the conserved β-sheet structure and were monomeric in solution (Fig. S3). Additionally, highly similar near-UV CD spectra suggest that the amino acid substitutions have only minor effects on the global tertiary structure of the antibody domain (Fig. S3).

Thermal unfolding experiments of the germline V_L_ domain constructs containing the individual patient mutations showed the largest stability decrease for the G94A mutant with a T_m_ value of 50.5 °C and the second largest effect for the G49R mutant with a transition temperature of 52.2 °C. The thermal stabilities of the remaining mutants Y31S, Y48F, and N51S were only slightly decreased with transition midpoints temperatures of 56.1 °C, 55.4 °C, and 54.4 °C, respectively (Table 1, Fig. S3). In the case of chemical unfolding, the strongest decrease in stability was observed for the G49R mutant with a C_m_ value of 2.91 M urea, whereas the G94A variant unfolded at a concentration of 3.25 M urea. Both the G49R and G94A variant show comparable $\Delta G_{un}^{app}$ values of 16.49 kJ/mol and 16.18 kJ/mol, respectively (Table S1). Again, the transition midpoints of the Y31S (4.2 M) and Y48F (4.15 M) mutants lie only slightly below that of the germline reference, while the N51S variant unfolded at 3.44 M urea (Table 1, Fig. S3).

Among the five single mutations, G49R and G94A exerted the strongest destabilizing effect on the germline V_L_ domain. Since G94A is a small, conservative mutation located in the hypervariable CDR3 loop, these results were unexpected. Therefore, we created double mutants by individually combining G94A with the remaining four mutations yielding the double mutations Y31S/G94A, Y48F/G94A, G49R/G94A, and N51S/G94A. The largest effect on thermal stability was observed for the double mutants G49R/G94A and N51S/G94A. These exhibited severely decreased thermal stabilities with melting temperatures of 47.0 °C and 48.6 °C, respectively. Accordingly, also in terms of chemical stability, the mutations G49R/G94A and N51S/G94A had the most significant effect with transition midpoints of 2.09 M and 2.56 M urea, respectively (Table 1, Fig. S3).

**Figure 3:**
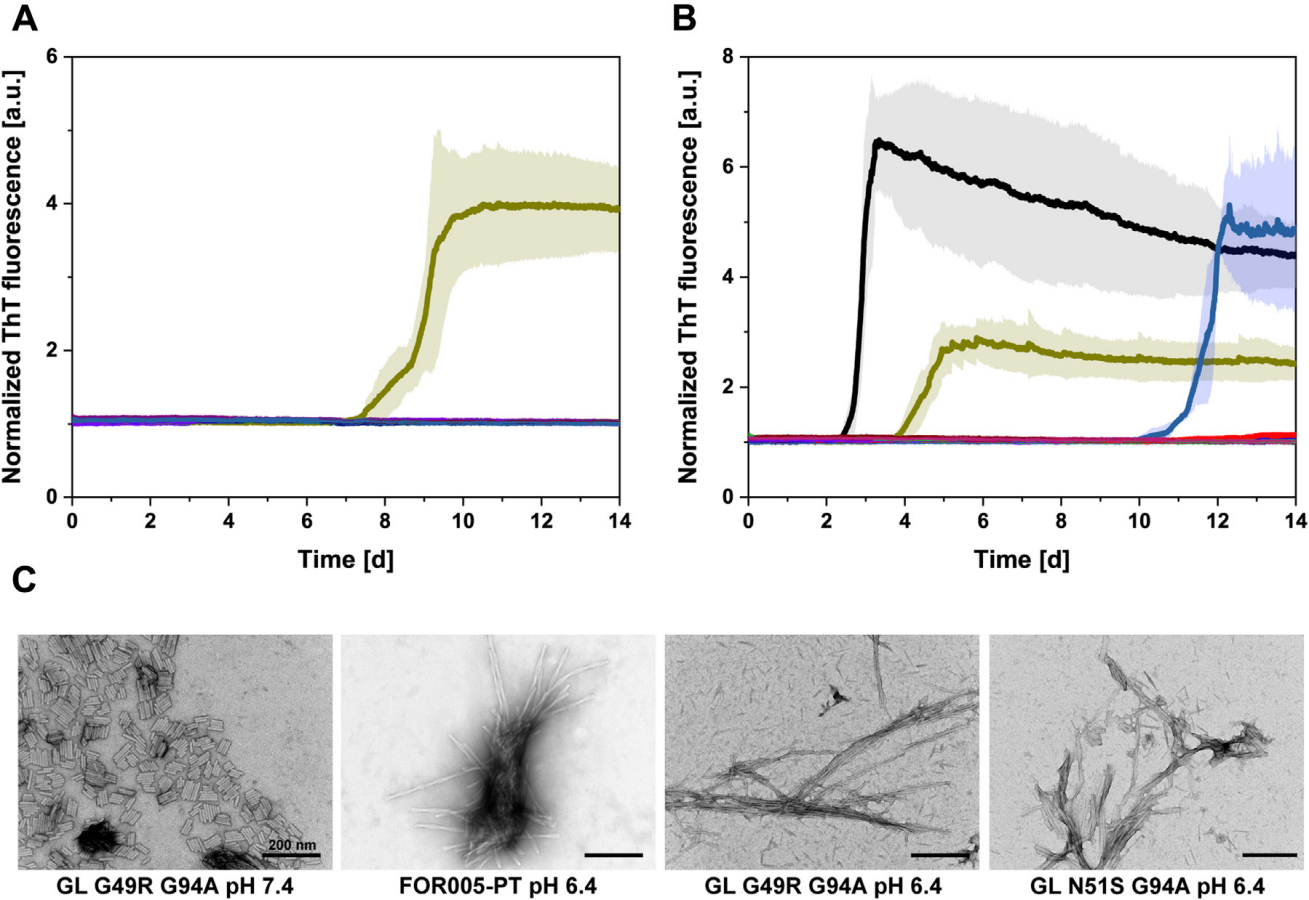
**The effects of point mutations on fibril formation propensity.***A*, fibril formation kinetics at 37 °C, pH 7.4, and continuous shaking show that G49R/G94A (*dark yellow*) is the only one of the nine investigated mutants that forms amyloid fibrils *in vitro*. All ThT kinetics were normalized to a fluorescence start value of 1. *B*, at pH 6.4, fibril formation of FOR005-PT (*black*) and G49R/G94A (*dark yellow*) is accelerated and also amyloid aggregation of N51S/G94A (*pale blue*) can be observed after approximately 10 days. Despite strong thermodynamic destabilization, N51S/G94A needs additional acidic conditions to form fibrils. *C*, TEM micrographs of all FOR005 variants were obtained after 2 weeks of incubation at 37 °C, pH 7.4 or 6.4, and continuous shaking in a Tecan Genios Platereader. Samples were stained using uranyl acetate. The four panels show the only samples that exhibited an increase in ThT fluorescence signal in the ThT assays depicted in *A* and *B*. The scale bar represents 200 nm.

Furthermore, ThT-binding kinetics and TEM micrographs revealed that G49R/G94A is the only mutant that forms amyloid fibrils *in vitro* at pH 7.4 and 37 °C (Fig. 3*A*). The N51S/G94A mutant, however, did not form fibrils within 2 weeks at pH 7.4, despite exhibiting significantly decreased thermodynamic stability, similarly to G49R/G94A. It has been shown that destabilization is not necessarily the only driving force in the amyloid formation pathway and that protein dynamics and population of nonnative intermediate states can play important roles, too (26, 27, 52). To further investigate the involvement of these molecular traits, additional fibril formation assays were performed at pH 6.4, since acidification can lead to a decrease in stability and population of alternatively folded intermediate states (53, 54, 55). As expected, in ThT assays carried out at pH 6.4, fibril formation was accelerated for FOR005-PT and G49R/G94A, but also for N51S/G94A fibril formation was observed after approximately 10 days (Fig 3*B*). The presence of amyloid fibrils in the ThT assay was confirmed by TEM micrographs (Fig. 3*C*). These findings imply that the amyloid aggregation of FOR005-PT relies on a mechanism in which domain destabilization is an important, yet not the only decisive biophysical factor.

Overall, the results for the single and double mutations show that out of the five mutations two are mainly responsible for the significant loss in thermodynamic stability and the gain in amyloid formation propensity. Remarkably, the conservative G94A mutation in the exposed CDR3 loop has a strong impact on the biophysical properties of the V_L_ domain, despite being located in the most variable part of the protein.

### Conformational dynamics are linked to decreased protein stability and amyloid formation

Previous studies have demonstrated that there is a causal link between conformational dynamics and aggregation propensity, as well as cellular toxicity of prefibrillar species (27, 56, 57, 58). Therefore, we set out to investigate the structural dynamics and flexibility of patient and germline V_L_ domains by limited proteolysis and hydrogen/deuterium exchange mass spectrometry (H/DX-MS).

**Figure 4:**
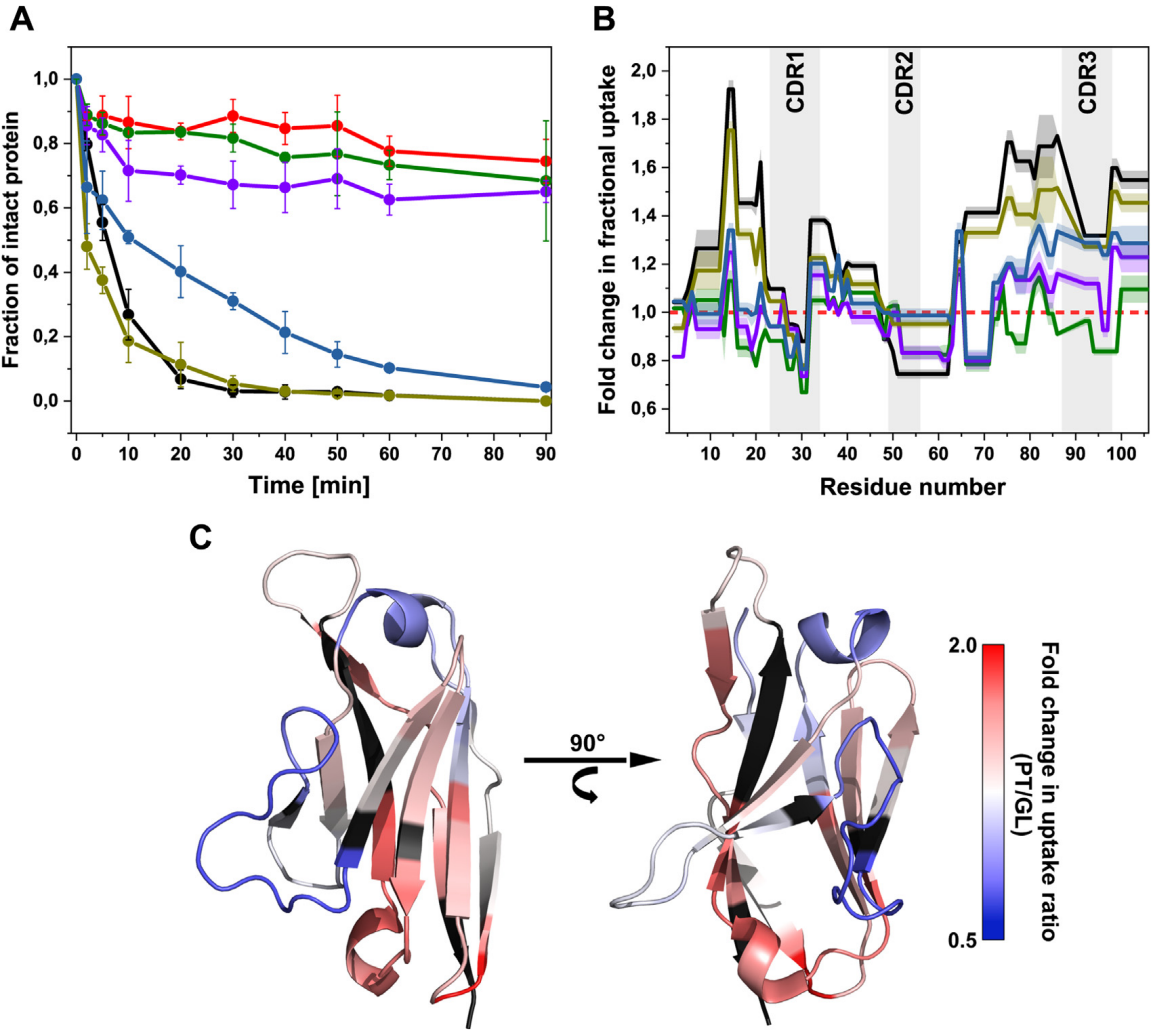
**Conformational dynamics play a major role in the fibril formation of FOR005-PT.***A*, limited proteolysis of FOR005 constructs with trypsin was carried out in triplicates at room temperature using a protein/protease ratio of 15/1 (w/w). FOR005-PT is shown in *black*, GL in red, G49R in *green*, G94A in *violet*, G49R/G94A in *dark yellow*, and N51S/G94A in *pale blue*. Increased susceptibility to proteolytic degradation implies enhanced structural dynamics. *B*, fractional deuterium was detected after 2 h incubation with D_2_O by ESI-TOF/TOF mass spectrometry to give peptide-resolved information on protein backbone dynamics. The fold change in fractional uptake compared to the germline V_L_ was calculated by dividing the uptake values of the respective mutants by the uptake values of FOR005-GL. Therefore, a fold change value below 1 means lower flexibility than the germline, a value above 1 indicates enhanced dynamics in comparison. The data sets for the mutants are colored according to *A*. The *dashed red line* at a value of 1 represents the germline V_L_. *C*, the fold change in uptake of FOR005-PT was plotted onto the crystal structure of the patient V_L_ domain. *Red color* indicates strongly enhanced dynamics in the patient-derived V_L_ domain, *blue color* indicates increased dynamics of the germline protein. Residues colored in *black* could not be analyzed in the H/DX-MS experiments. The most strongly affected segments lie in the β-sheet framework, especially in structural regions close to the C terminus of the V_L_ domain.

Limited proteolysis allows obtaining information about structural flexibility since proteolytic degradation is increased due to enhanced protein dynamics and local unfolding (59). When we carried out limited proteolysis experiments with the proteases trypsin or proteinase K, we found that the patient-derived V_L_ domain was degraded much faster than its germline counterpart pointing toward a higher degree of conformational dynamics (Fig. 4*A*, Fig. S5). Further, the single-point mutants G49R and G94A behave similarly to the germline V_L_ domain and exhibit overall slow degradation kinetics. However, G94A is processed faster and to a greater extent than G49R and FOR005-GL. The double mutant N51S/G94A is also cleaved much faster than the germline and the observed single mutants, yet not as fast as the patient-derived V_L_ domain. Interestingly, the double mutant G49R/G94A is degraded even more readily than the patient V_L_ domain FOR005-PT (Fig. 4*A*, Fig. S5).

H/DX-MS was applied to gain more detailed insights into the conformational dynamics of both the patient and germline V_L_ domain. This method is based on the enhanced solvent exchange rates of backbone amide hydrogens in flexible protein regions from which peptide-resolved dynamic information can be derived after pepsin cleavage and mass spectrometric analyses (60). Fractional deuterium uptake was determined for FOR005-PT, FOR005-GL, G49R, G94A, as well as the double mutants G49R/G94A and N51S/G94A. The fold change in fractional uptake was calculated by dividing uptake ratios of the investigated mutants by the uptake ratios of the germline V_L_ domain (Fig. 4*B*). A value below 1 indicates that the germline exhibits higher deuterium uptake, whereas a value above 1 shows increased deuterium uptake for the observed mutant. Conformational dynamics are especially pronounced for residues 12 to 20, residues 65 to 85, and residues 97 to 105 in the case of FOR005-PT and for the double mutant G49R/G94A (Fig. 4*B*). The double mutation, however, still exhibits slightly lower flexibility compared with the actual patient V_L_ that contains all five substitutions. Interestingly, residues 50 to 60 including the CDR2 loop are more dynamic in the germline V_L_ domain and in G49R/G94A. The double mutant N51S/G94A also exhibits lightly increased dynamic behavior, especially for residues 80 to 105, whereas the single mutants G49R and G94A, in comparison, do not impose a strong increase in conformational flexibility. Notably, G94A has a slightly larger effect on overall dynamics than G49R (Fig. 4*B*). To better visualize which parts of the patient-derived V_L_ domain experience enhanced dynamics in comparison with the germline, the change in fractional uptake was plotted onto the crystal structure of FOR005-PT (PDB: 5L6Q). Structurally, the most affected regions correspond to β-strands A2 and B and the small loop connecting them (residues 12–20), β-strands E and F including the small helical segment between them (residues 65–85), and the C-terminal β-strands G1 and G2 (Fig. 4*C*). In summary, our results show that there is a clear connection between conformational dynamics and amyloid aggregation. Remarkably, we observe the strongest increase in dynamics in conserved framework regions rather than the segments where the point mutations are located. These findings suggest that small mutation-induced changes in CDR loop conformations might propagate through the entire domain architecture and thereby lead to increased dynamics in framework regions, which causes lower stability and enhanced aggregation propensity.

### Unfavorable main chain conformations in the CDR2 and CDR3 loops destabilize the V_L_ domain

**Figure 5:**
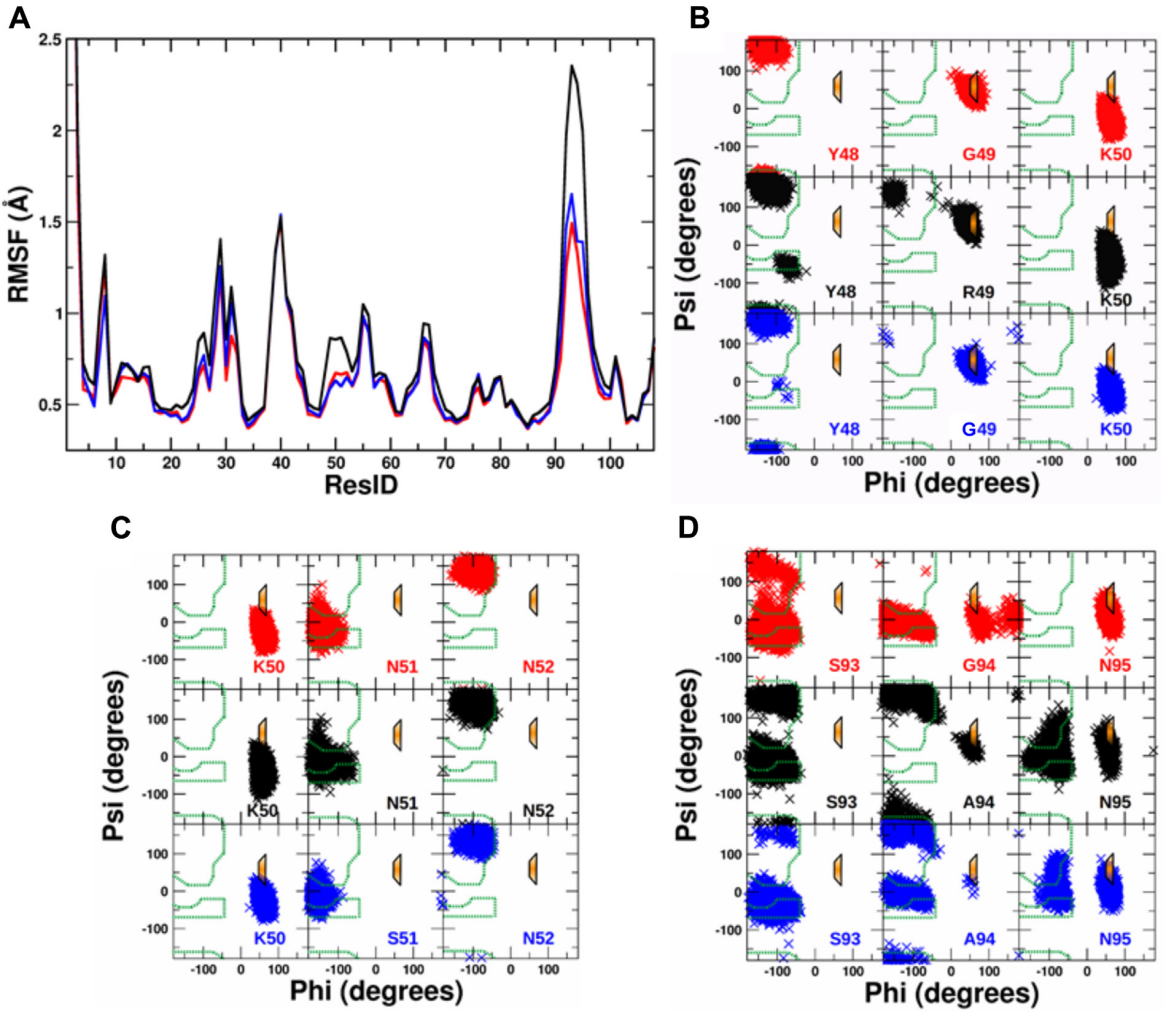
**MD simulations show energetically unfavorable backbone conformations in CDR2 and CDR3.***A*, root-mean-square fluctuations (RMSF) observed in MD simulations (1 μs, at 310 K) along the residue sequence for the FOR005-GL V_L_ variant (*red line*), the FOR005-GL G49R/G94A (*black line*), and the GL N51S/G94A (*blue line*) substitutions. *B*, sampled backbone dihedral angles phi and psi plotted as Ramachandran plots for residues 48 to 50 (same color code as in *A*). Favorable regions for non-Gly residues are indicated by a *green dashed boundary* in the Ramachandran plots and a regime favorable for Gly but less for other amino acids is indicated in *orange* with a *blue boundary*. *C*, same as in *B* but for residues 50 to 52. *D*, same as in *B* but for residues 93 to 95.

To gain further insight into the flexibility of the patient V_L_ domain, molecular dynamics (MD) simulations were performed in explicit solvent on the FOR005-GL, the GL G49R/G94A, the GL N51S/G94A, the FOR005-PT V_L_ and a PT variant containing the R49G and A94G double substitution. On the timescale of 1 μs, the variants were stable during the MD simulations and exhibited only small and similar deviations from the start structure (Fig. S6). Comparison of root-mean-square fluctuations (RMSF) indicated the lowest fluctuations for FOR005-GL, slightly enhanced fluctuations for GL N51S/G94A, and significantly increased fluctuations especially around residue 49 and 94 in case of the GL G49R/G94A variant (Fig. 5*A*). Slightly larger conformational fluctuations on the MD timescale were also observed for the FOR005-PT variant compared with the PT R49G/A94G mutation (Fig. S6*A*).

The inspection of the peptide backbone dihedral angles in the loop regions near residue 49 and 94 revealed the sampling of the left-handed helical regimes in the Ramachandran plots of residues 49 and 50 as well as 94 and 95 (but not for residues 51 or 52, Fig. 5, *B*–*D*). This regime is sterically favorable in case of the glycine but less so for nonglycine residues. Hence, R49 or A94 creates steric strain in the loop structure, whereas G49 or G94 relaxes this strain. Also, in the case of FOR005-PT, the MD simulations reveal sampling of sterically unfavorable peptide backbone states that—in a relaxed peptide structure—are typically only adopted by glycine residues (Fig. S6*B*). Notably, these unfavorable backbone states are also observed in the crystal structure of the patient protein. Hence, in the patient structure, the loop forces its residues at least partially into an energetically unfavorable backbone structure upon folding. The “germline” substitutions R49G and A94G can relax this strain because now the glycine residues at positions 49 and 94 are better compatible with the required backbone structure. Interestingly, the substitutions can also have an effect on neighboring residues and partially modify their backbone sampling (Fig. 5, *B*–*D*, Fig. S6, *B* and *C*). Energetically, the stress of enforcing unfavorable backbone conformation of residues 49 and 94 can amount to several kcal/mol and hence could be the reason for the much lower stability of FOR005-PT and of the variants with nonglycine residues at positions 49 and 94. For residues 51 and 52 approximately the same sampling of favorable backbone states was observed (Fig. 5*C*) with no significant effect of the N51S substitution.

## Discussion

Systemic LC amyloidosis is a highly complex protein misfolding disease because of the enormous sequence variability of the soluble precursor protein—the antibody LC. This renders the mechanistic understanding of the amyloid aggregation process a very challenging task. Different, case-dependent organ involvement and a wide spectrum of symptoms further complicate analysis and treatment of this rare disorder (3, 4). Additionally, there are a number of other factors that can affect fibril formation, disease onset, and progression, including proteolytic processing of the precursor LCs (7, 10).

Whether proteolytic processing of LC precursors is a prerequisite or a consequence of amyloid formation still remains enigmatic (12, 14, 30, 61). It has been shown that the C_L_ domain can exert a protective function *in vitro* and that full-length LCs do not readily aggregate into amyloid fibrils (56, 62, 63). In the case of FOR005, the V_L_ domain was identified as the sole component of amyloid deposits in the patient’s tissue (36). Accordingly, *in vitro* only the patient-derived V_L_ domain but not the corresponding full-length LC formed amyloid fibrils, thus implying a protective role of the C_L_ domain.

FOR005 is an interesting case, as the V_L_ domain contains four CDR mutations and only one framework mutation compared with its germline counterpart. Of note, the exact location of the CDRs depends on which domain numbering system is used. The three systems according to Kabat, Chothia, and IMGT are the most common ones (38, 43, 44). When the IMGT numbering scheme is applied to FOR005, the substitution N51S would be considered a framework mutation rather than a CDR2 mutation. However, the Kabat and Chothia classifications identify this residue as belonging to the CDR and this coincides with the Consurf residue conservation analysis (Fig. S1). Furthermore, the identification of a suitable germline sequence for a given V_L_ domain can yield different results depending on which method/database is used. We applied abYsis, IgBLAST, and IMGT to identify a V_L_ domain with highest possible amino acid sequence identity (38, 39, 40). The most important practical test for the germline sequence of choice is whether it forms fibrils, as this allows to identify and test the effect of the patient mutations concerning their amyloidogenic potential (56).

Up to now, mostly framework mutations have been reported as key factors in LC amyloid aggregation (22, 24, 26, 56, 62, 64, 65, 66, 67, 68). Regarding the only framework mutation in FOR005-PT—Y48F—it has already been shown for a different V_L_ domain that this particular mutation has little to no influence on domain stability and aggregation propensity (22). Therefore, we hypothesized that only the CDR mutations play a crucial role in the case of FOR005, which was confirmed by the experimental results. CDR loops are not only involved in antigen recognition, they have also been shown to play important structural roles in antibody domain architecture and V_H_/V_L_ domain association. Various experimental and computational studies on V_L_ domains demonstrated that CDRs can have a strong influence on the folding pathway, stability, and conformation of the protein (69, 70, 71, 72). The involvement of a CDR mutation in LC amyloidogenicity has been shown for a proline residue in the CDR3 loop of an amyloidogenic V_L_ domain. Its deletion resulted in enhanced stability and delayed fibril formation kinetics (73). Furthermore, nonconservative mutations in the V_L_ domains of AL and multiple myeloma (MM) patients—also encompassing the CDR3 loops—were reported to affect the kinetic stability of the LCs (74). Additionally, it has been shown that CDR1 can act as a hotspot for aggregation and that a peptide based on part of a CDR3 segment can drive amyloid fibril formation due to enhanced steric zipper propensity (75, 76). However, a detailed mechanistic understanding of the effects caused by specific CDR residues in the context of the disease is still lacking (77).

Multiple studies on substitutions in the V_L_ domain demonstrate that misfolding and amyloid aggregation depend on the thermodynamic/kinetic stability, structural dynamics or partial unfolding, LC dimerization, and local conformational alterations of the native fold (10, 24, 27, 28, 29, 78). Thermodynamic and kinetic stabilities have widely been thought of as the major driving force in the misfolding and aggregation pathway (26, 56, 58, 79). In the case of FOR005, a synergistic combination of thermodynamic destabilization and altered conformational dynamics appears to determine the pathway of the soluble V_L_ monomer toward amyloid fibrils. FOR005-PT and FOR005-GL show a pronounced difference in stability with a ΔT_m_ of 12.8 °C and the mutations G49R and G94A have the largest impact on domain stability. However, fibril formation kinetics and thermodynamic data of the FOR005 double mutants suggest that destabilization through CDR mutations is not the only driving force in the amyloid formation process, since the severely destabilized N51S/G94A mutant only forms fibrils after prolonged incubation at lower pH. Additionally, an increase in conformational dynamics—mediated by the two decisive CDR mutations G49R and G94A—is necessary to induce the amyloid aggregation. Remarkably, the strongest increase in dynamics is observed in conserved protein core regions rather than the loop segments in which the mutations are located. MD simulations indicate that the loop residues 49, 50, 94, and 95 sample mostly backbone conformations that are energetically unfavorable for nonglycine residues, which lowers the overall stability of the folded structure. This coincides with reports that glycine is structurally preferable at positions with certain φ/ψ angles (22, 53). Hence, the interplay of the CDRs with the framework enforces an energetically unfavorable conformation of the loops. These strained loop structures affect framework dynamics and are the likely reason for the lower stability of variants with a nonglycine residue at the corresponding positions.

This is at first glance counterintuitive, as one might assume that the basic traits of CDRs are their sequence diversity and conformational flexibility that allow them to adapt to the structure of the antigen upon interaction. However, five out of the six CDRs in an antibody F_ab_ only adopt a limited number of backbone conformations, known as canonical classes, with the heavy chain CDR3 (CDRH3) being the only exception (43, 80, 81, 82). Therefore, it seems plausible for some CDR mutations in LCs to induce unfavorable loop conformations, which represent a deviation from the canonical CDR class and thereby put structural strain on the framework. This deviation can be seen by aligning the crystal structure of the amyloid-forming FOR005-PT with the structures of similar, nonamyloidogenic V_L_ domains (Fig. S7). A similar canonical class alteration has been observed for the CDR1 loop of some amyloidogenic λ6 LCs (83). Nonetheless, especially conservative mutations in exposed loops were not expected to drastically alter protein structure and stability (33). Yet, the CDR mutations in FOR005—especially the conservative G94A substitution—strongly affect V_L_ domain stability and conformation. The changes caused by unfavorable CDR loop conformations seem to propagate through the entire protein inducing increased flexibility, which leads to the enhanced population of partially unfolded, aggregation-competent states (52, 78). Therefore, an altered interplay of hypervariable loops and conserved framework can play a key role in stability and amyloidogenicity of V_L_ domains (69, 73). In this context, FOR005-PT represents the first case where the onset of fibril formation is directly and mechanistically correlated to the substitution of two distinct amino acids in CDR loops. Surprisingly, one of these two decisive substitutions is the small, conservative G94A mutation in the surface-exposed CDR3 loop.

Pradhan *et al.* (19) have recently shown that the R49 residue in FOR005-PT plays a key role in stabilizing the fibril core. With this information, it becomes plausible that mutations in amyloid-forming LCs can serve different purposes. The G94A mutation leads to a conformational change in the CDR3 loop, which thereby adopts a structure that differs from the canonical CDR class. This conformational change results in enhanced framework dynamics and decreased overall domain stability. To illustrate this concept, a structural alignment of FOR005-PT was performed with three highly similar, nonamyloidogenic V_L_ domains taken from the PDB (Fig. S7). The mutation-induced changes in CDR loop conformation depict the described deviation from the canonical CDR class. In the final core structure of FOR005-PT fibrils, however, A94 does not play an important role. Seemingly, its only effect lies in the destabilization of the precursor V_L_ domain. The CDR2 mutation G49R, on the other hand, drives amyloidogenesis both by altering CDR2 loop conformation and by providing a stabilizing side chain interaction in the fibril core (19). Yet, as our data show G94A mediates a larger increase in conformational dynamics than G49R, especially in the framework 3 region and the C-terminal part of the domain (Fig. 4). Further, the CDR2 mutation N51S is also capable of inducing fibril formation. Thus, the primary role of G49R in the fibril formation pathway of FOR005-PT appears to lie in stabilizing the final product of the pathway—the core of the amyloid fiber. Individually, however, the two point mutations do not induce fibril formation *in vitro*. Yet in combination, the two CDR mutations G49R and G94A act synergistically as the obtained stabilities and apparent free energies imply (Table 1, Table S1). In summary, the decisive, amyloid-driving mutations are not necessarily involved in propagating fibril formation by providing specific side chain interactions within the fibril structure. Rather, they destabilize the V_L_ domain in a specific way, increasing the dynamics of framework regions, which upon structural transitions form the conformationally rearranged fibril core. Thus, the relationship of the mutations and fibril formation can be topologically indirect as seen by the effects of the G94A mutation in FOR005.

In conclusion, our findings add further proof to the concept that thermodynamic stability is an important, yet not the only crucial molecular determinant in the fibril formation pathway of LCs and that conformational dynamics play an important part. Additionally, we show that different mutations can be important in amyloid formation by either destabilizing the precursor protein or stabilizing the final fibril core structure or even both. Furthermore, our study provides detailed mechanistic information on the limitations of CDR flexibility, on antibody domain architecture, and how mutations in the hypervariable CDRs can have a major impact on V_L_ domain integrity and induce fibril formation.

## Experimental procedures

All chemicals were purchased from Sigma-Aldrich or VWR unless stated otherwise.

### Sequence and structure analysis

The cDNA sequence of FOR005-PT was previously reported by Annamalai *et al*. (36) (https://www.ncbi.nlm.nih.gov/nuccore/KX290463). The corresponding germline sequence, FOR005-GL, was determined using IgBLAST (https://www.ncbi.nlm.nih.gov/igblast/), the international immunogenetics information system (http://www.imgt.org/), and the abYsis database (http://www.abysis.org/abysis/). The GenBank accession code for the germline V_L_ domain is AAZ13705.1. For bioinformatic analyses of the protein sequences and structures Clustal Omega (84), Consurf (45), AmylPred2 (46), MetAmyl (47), ZipperDB (48), and SWISS-MODEL (49) were used.

### Cloning, mutagenesis, protein expression, and purification

Synthetic DNA constructs of FOR005-PT/GL and FOR005-PLC/GLC in pET28b(+) were obtained from Invitrogen. Variants were produced by site-directed mutagenesis using primers designed with NEBaseChanger. Primers were synthesized by Eurofins Genomics. Q5-Polymerase chain reactions and subsequent KLD enzyme reactions were performed according to the manufacturer's protocol. Plasmid sequencing was performed by Eurofins Genomics. Plasmids were transformed into *E.coli* BL21 (DE3)-star cells and the proteins were expressed as insoluble inclusion bodies at 37 °C over night after induction with 1 mM IPTG. Cells were harvested and inclusion bodies prepared as previously described (85). Inclusion bodies were solubilized in 50 mM Tris/HCl, 8 M urea, 0.1% β-mercapto ethanol, pH 8.0 at room temperature for 4 to 8 h and then dialyzed against an excess of 50 mM Tris, 5 M urea, pH 8.0 at 10 °C over night. The solubilized protein was then subjected to anion exchange chromatography using Q-Sepharose (GE Healthcare, Uppsala, Sweden). Protein-containing fractions were pooled and diluted to 0.5 mg/ml protein or below. The diluted protein was dialysed against an excess of 50 mM Tris, 3 M urea, pH 8.5 at 10 °C over night. Afterwards, the protein was dialyzed against PBS pH 7.4 for approximately 24 h at 10 °C. As a polishing step, the refolded protein was purified by size-exclusion chromatography using a Superdex75 column (GE Healthcare, Uppsala, Sweden) running in PBS. Protein quality was checked by SDS-PAGE and ESI-ion trap mass spectrometry.

### Circular dichroism spectroscopy

CD measurements were carried out on a Chirascan spectropolarimeter (Applied Photophysics, Surrey, UK) and on a JASCO J-1500 CD spectrometer (JASCO). Far-UV spectra were recorded in a 1 mm quartz cuvette at 20 °C from 260 nm to 200 nm using 10 μM protein diluted in PBS. Near-UV spectra were recorded in a 2 mm quartz cuvette at 20 °C from 260 nm to 320 nm using 50 μM protein in PBS. Thermal transitions were recorded from 20 to 90 °C at 205 nm using a heating rate of 1 °C/min.

### Analytical ultracentrifugation

For AUC measurements, a ProteomLab XL-I centrifuge (Beckman) equipped with absorbance optics was used. The protein concentration for the measurements was 40 μM in PBS. The assembled cells were loaded with 350 μl of sample solution. The cells are equipped with quartz windows and 12-mm-path-length charcoal-filled epon double-sector centerpieces. An eight-hole Beckman-Coulter AN50-ti rotor was used for all measurements, which were carried out at 42,000 rpm and 20 °C. Sedimentation was continuously scanned with a radial resolution of 30 mm and monitored at 280 nm. For data analysis, SEDFIT with continuous c(S) distribution mode was used (86, 87).

### Fluorescence spectroscopy

Reversibilty of unfolding was checked by incubating 10 μM native patient and germline V_L_ domain (each in triplicates) with 6 M urea for 2 h at room temperature. Then the samples were diluted 1:9 with PBS pH 7.4 for over night refolding yielding a final protein concentration of 1 μM and a final urea concentration of 0.6 M. For comparison, 1 μM native V_L_ domains were incubated with 0.6 M and 6 M urea for 24 h. Fluorescence spectra were recorded at 25 °C on a Horiba FluoroMax4 spectrofluorimeter (Horiba Jobin Yvon) with an excitation wavelength of 280 nm and emission from 300 to 400 nm. Excitation and emission slits were set to 5 nm, and for every spectrum two accumulations were averaged.

Chemical unfolding transitions were carried out in triplicates by incubating 1 μM of protein with increasing concentrations of urea in a sample volume of 200 μl in reaction tubes. After incubation over night at room temperature, the samples were transferred into a 96-well Greiner UV-star plate (Greiner Bio-One, Kremsmünster Austria) and intrinsic tryptophan fluorescence was monitored at 25 °C in a Tecan Infinite M Nano+ plate reader (Tecan Group Ltd). The excitation wavelength was 280 nm and emission spectra were recorded from 300 to 400 nm. Transition curves were obtained by plotting normalized fluorescence intensities at the wavelength at which native and unfolded state shows the largest signal difference against the concentration of urea. The transition curves represent triplicates that were averaged and normalized. Subsequently, data was analyzed with Origin by applying a Boltzmann fit and a two-state unfolding fit model to obtain $\Delta G_{un}^{app}$ and cooperativity values (88).

### Thioflavin T-binding kinetics

Prior to sample preparation, protein stock solutions were centrifuged in an Optima MAX-E ultracentrifuge (Beckman) for 3 to 4 h at 40,000 rpm in order to remove aggregates. Additionally, all assay components were filtered through a 0.22 μm filter (Merck) before the samples were prepared. For all measurements, 200 μl of each sample was incubated in 96-well Nunc plates (Nunc, Thermo Fisher) sealed with Crystal Clear PP sealing foil (HJ-Bioanalytik GmbH). Thioflavin T assays were carried out in triplicates with 15 μM protein, 7.5 μM ThT, 0.05% sodium azide, pH 7.4 or 6.4, at 37 °C under continuous orbital shaking in a Tecan Genios platereader with the shaking intensity set to high (Tecan Group Ltd). For determining the ThT fluorescence of the samples, the excitation wavelength was 440 nm, the emission wavelength was 480 nm, and the gain was set to 70 to 75. Values of midpoint amyloid fibril formation (t_50_) were determined using a Boltzmann fit.

### Transmission electron microscopy

Activated copper grids (200 mesh) were loaded with 10 μl of sample from finished ThT assays for 1 min. The grids were washed with 20 μl H_2_O and stained with 8 μl of a 1.5% uranyl acetate solution for 1 min. Excess solutions were removed from the grids with filter paper. TEM micrographs were recorded at 120 kV on a JEOL JEM 1400-plus transmission electron microscope (JEOL Germany GmbH).

### Limited proteolysis

The V_L_ domains were diluted to 0.3 mg/ml in 100 mM Tris, 100 mM NaCl, 10 mM CaCl_2_, pH 7.8 and incubated at room temperature with trypsin using substrate/enzyme ratio of 15/1 (w/w) or with proteinase K at a substrate/enzyme ratio of 150/1 (w/w). At defined time points, samples were taken from the reaction and mixed with PMSF (final concentration 2 mM) and Lämmli buffer to stop the proteolytic degradation. Afterward, the samples were run on a SERVA Prime 4 to 20% SDS gel, and protein ratios were subsequently analyzed using NIH ImageJ (89).

### Hydrogen/deuterium exchange mass spectrometry (H/DX-MS)

For all H/DX-MS experiments, a fully automated system equipped with a Leap robot (HTS PAL; Leap Technologies, NC), a Waters ACQUITY M-Class UPLC, an H/DX manager (Waters Corp), and a Synapt G2-S mass spectrometer (Waters Corp) were used as previously described (90). Protein samples with a concentration of 30 μM were diluted in a ratio of 1:20 with PBS buffer (pH 7.4) containing deuterium oxide. The samples were incubated with D_2_O for 0 s, 10 s, 1 min, 10 min, 30 min, or 2 h. The exchange was stopped by diluting the labeled protein 1:1 in quenching buffer (200 mM Na_2_HPO_4_ × 2 H_2_O, 200 mM NaH_2_PO_4_ × 2H_2_O, 250 mM Tris (2-carboxyethyl)phosphine, 3 M GdmCl, pH 2.2) at 1 °C. Proteolytic online digestion was performed using an immobilized Waters Enzymate BEH Pepsin Column (2.1 × 30 mm) at 20 °C. The resulting peptides were trapped and separated at 0 °C on a Waters AQUITY UPLC BEH C18 column (1.7 mm, 1.0 × 100 mm) by an H_2_O to acetonitrile gradient with both eluents containing 0.1% formic acid (v/v). Eluting peptides were directly subjected to the Synapt TOF mass spectrometer by electrospray ionization. Prior to fragmentation and mass detection, peptides were additonally seperated by drift time. Samples were pipetted by a LEAP autosampler (HTS PAL; Leap Technologies, NC). Data analysis was performed with the Waters Protein Lynx Global Server PLGs (version 3.0.3) and the DynamX (Version 3.0) software package.

### Molecular dynamics simulations

MD simulations were carried out and analyzed using the Amber18 simulation package (91). Simulations were performed starting from the FOR005-PT V_L_ variant for which a crystal structure is available (PDB: 5L6Q) and on the *in silico* generated variants with the R49G und A94G substitutions, the FOR005GL (wild-type sequence), the GL G49R/G94A, and the GL N51S/G94A sequence variants. Each protein was solvated in TIP3P water in a periodic octahedral box with a minimum distance of protein atoms to the box boundary of 10 Å (92). The ff14SB force field was employed and Na^+^ and Cl^−^ ions were added to neutralize the system and reach an ion concentration of 0.15 M. Energy minimization of each system was performed with the sander module of Amber18 (2500 minimization cycles). The systems were heated in steps of 100 K (50 ps per step) to a final temperature of 310 K with the solute nonhydrogen atoms harmonically restraint to the start structure. All bonds involving hydrogen atoms were kept at optimal length. In additional four steps, the harmonic restraints were removed stepwise. For the subsequent production simulations, hydrogen mass repartitioning (HMR) was employed allowing a time step of 4 fs (instead of 2 fs used during heating and equilibration). Unrestrained production simulations were extended to 1 μs for each system. Coordinates were saved every 8 ps. Root mean square deviation (RMSD), root mean square fluctuations (RMSF), and analysis of dihedral angle distributions were performed using the cpptraj module of Amber18.

## Data availability

All data are contained within the article.

## Conflict of interest

The authors declare no conflict of interest.
