# Supplementary material for: Molecular mechanism of amyloidogenic mutations in hypervariable regions of antibody light chains
Source: J Biol Chem. 2021 Jan 26;296:100334. doi: 10.1016/j.jbc.2021.100334 (PMC7949129; doi:10.1016/j.jbc.2021.100334)
Supplement: Supplemental Figures S1–S7 and Table S1 [file mmc1.pdf]

Molecular mechanism of amyloidogenic mutations in hypervariable regions of antibody light chains

Supporting information

Georg J. Rottenaicher[1], Benedikt Weber[1,3], Florian Rührnößl[1], Pamina Kazman[1], Ramona M. Absmeier[1], Manuel Hitzenberger[2], Martin Zacharias[2], Johannes Buchner[1*]

[1]Center for Integrated Protein Science Munich at the Department Chemie, Technische Universität München, Lichtenbergstr. 4, 85748 Garching, Germany

[2]Center for Integrated Protein Science Munich at the Physik-Department, Technische Universität München, Lichtenbergstr. 4, 85748 Garching, Germany

[3]Current address: Roche Diagnostics GmbH, Nonnenwald 2, 82377 Penzberg, Germany

* To whom correspondence should be addressed: johannes.buchner@tum.de

Content: Table S1, Fig. S1, Fig. S2, Fig. S3, Fig. S4, Fig. S5, Fig. S6, Fig. S7

Table S1: Apparent free energies obtained by chemical unfolding transitions at 25 °C. The data from urea unfolding transitions was fitted using a two-state model, although we showed that FOR005-PT and –GL unfold irreversibly under the stated conditions (24 hours at room temperature). Therefore, a two-state model can in principle not be applied to describe the unfolding of these V_L domains in terms of free energy. The values derived from the two-state fit represent merely apparent unfolding free energies.

V_L domain	ΔG_{un}^{app} kJ mol^{-1}
FOR005-PT	11.51 ± 0.63
FOR005-GL	19.53 ± 1.20
GL Y31S	21.42 ± 2.00
GL Y48F	19.37 ± 1.43
GL G49R	16.49 ± 0.36
GL N51S	17.08 ± 0.79
GL G94A	16.18 ± 3.23
GL Y31S/G94A	16.48 ± 1.86
GL Y48F/G94A	17.11 ± 0.81
GL G49R/G94A	14.14 ± 0.84
GL N51S/G94A	15.45 ± 0.81

Figure S1

A

B

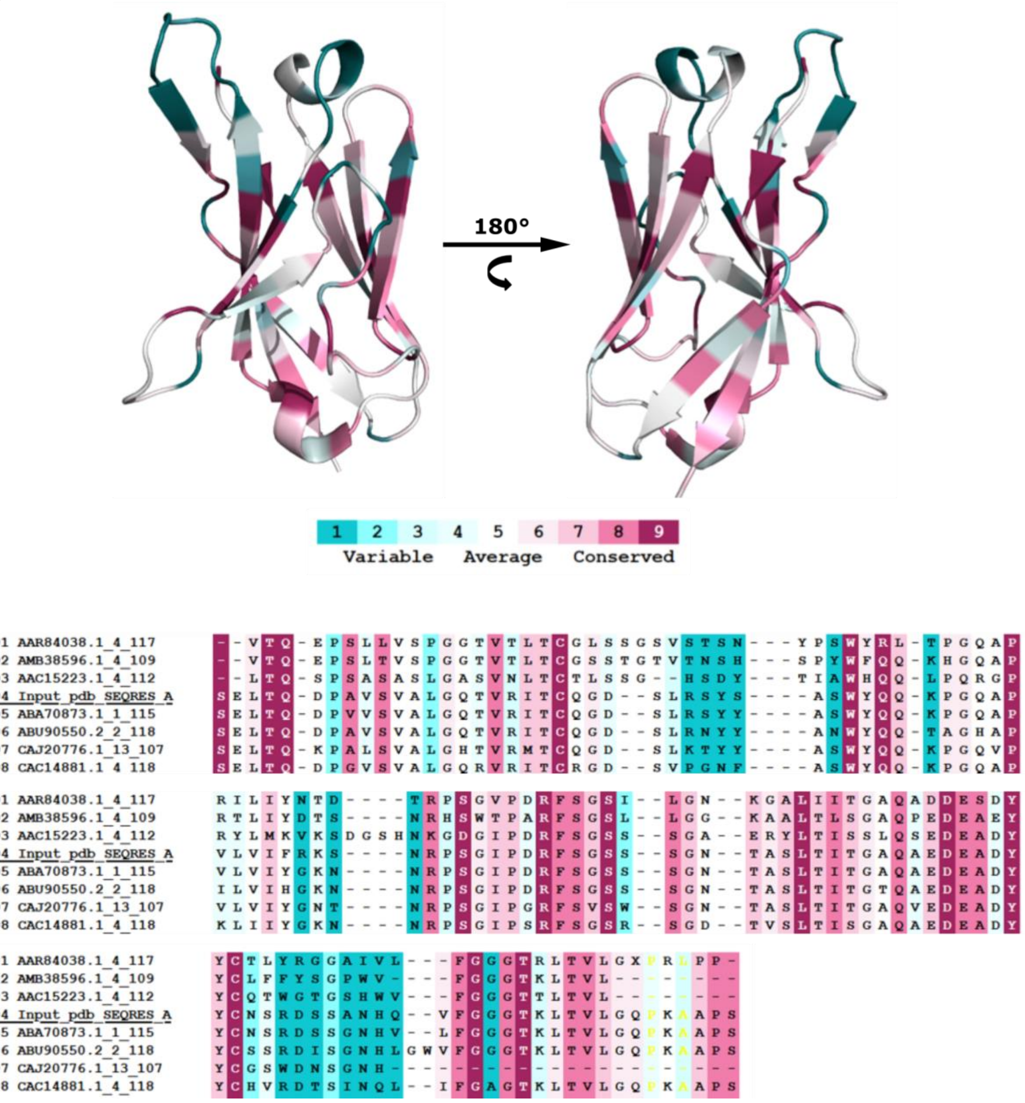

Figure S1: Residue conservation in FOR005-PT analyzed with Consurf.

A) Color-coded conservation scores derived from Consurf were plotted onto the crystal structure of FOR005-PT. Highly variable residues with a conservation score of 1 are shown in dark cyan, highly conserved positions with a score of 9 are shown in purple.

B) Outtake of the Consurf multiple sequence alignment comprising eight sequences including the sequence of FOR005-PT (“004_Input_pdb_SEQRES_A”).

Figure S2

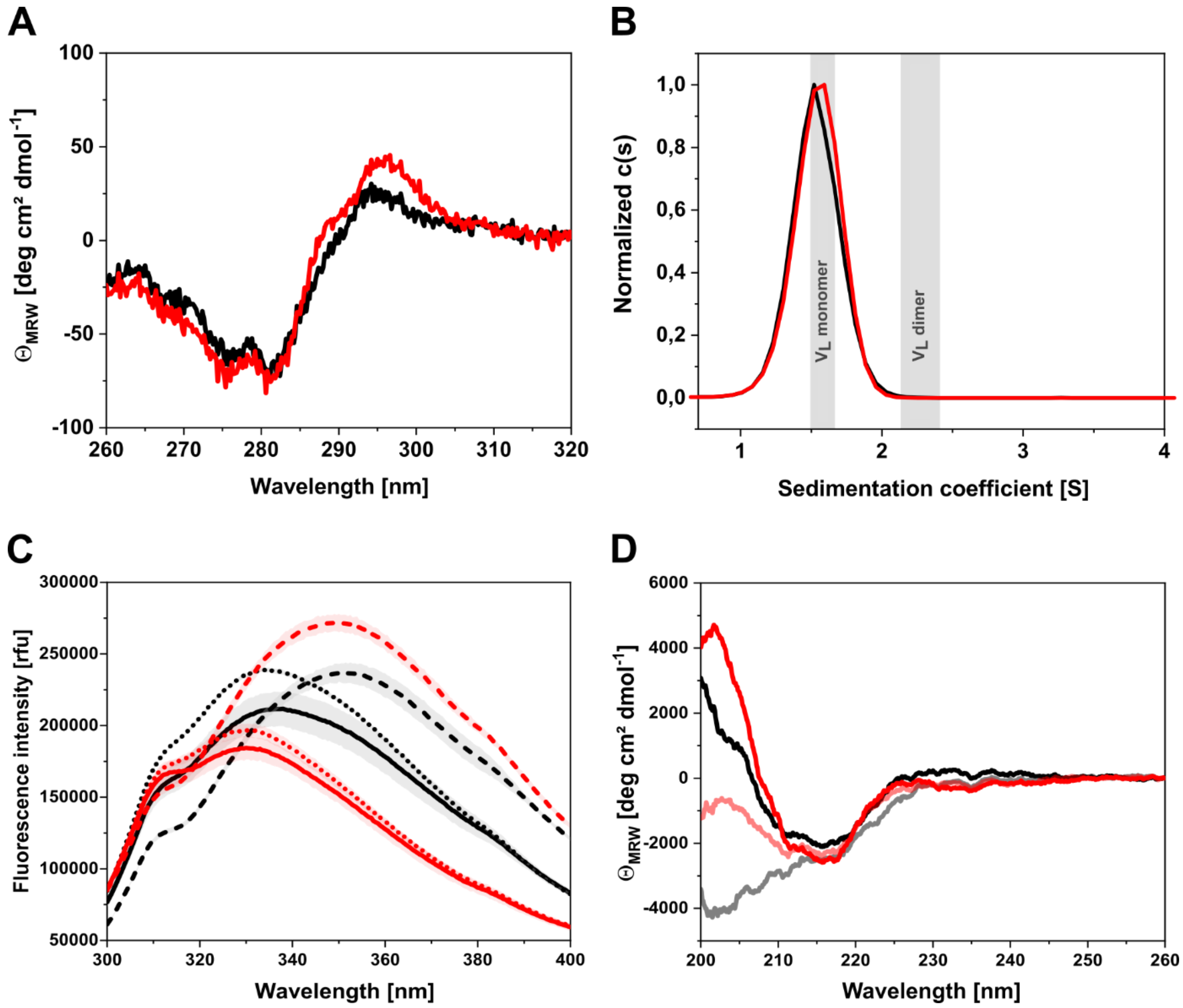

Figure S2: Biophysical characterization of FOR005-PT and GL.

A) Near-UV CD spectra of FOR005-PT (black) and -GL (red) were recorded at 20 °C with 50 µM protein in a 2 mm quartz cuvette. Near-UV circular dichroism reports on the tertiary structure fingerprint of a protein.

B) Analytical ultracentrifugation of PT (black) and GL (red) shows that both proteins are monomeric in solution. An S-value of 1.5 - 1.6 Svedberg corresponds to a V_L monomer with an estimated molecular weight of around 12 kDa (according to SEDFIT). A V_L-V_L homo dimer would appear at an S-value of 2.2 - 2.4 Svedberg corresponding to an estimated molecular weight of approximately 24 - 25 kDa (indicated by grey bars).

C) Fluorescence spectra of folded (straight line), completely unfolded (dashed line), and refolded (pointed line) FOR005-PT (black) and FOR005-GL (red) were recorded in triplicates at a protein concentration of 1 µM using a 1 cm fluorescence quartz cuvette. For the folded spectra, the native V_L domains were equilibrated with 0.6 M urea 24 hours at room

temperature, for the unfolded spectra they were equilibrated with 6 M urea. For refolding, the native V_L domains were first unfolded for two hours at room temperature using 6 M urea and subsequently diluted to 0.6 M urea using PBS. The refolded samples were then equilibrated for 24 hours at room temperature prior to measurement. The difference of folded and refolded spectra shows the irreversibility of chemical unfolding for FOR005 variants. However, the difference between folded and refolded spectra is more pronounced for the patient V_L domain suggesting a higher refolding propensity for the germline variant.

D) Far-UV CD spectra of native FOR005-PT and FOR005-GL at 20 °C are shown in black and red, respectively. The samples were heated up to 90 °C with a heating rate of 1 °C/min and subsequently cooled down back to 20 °C. Far-UV spectra were again recorded after over night equilibration at 20 °C and are shown in grey for FOR005-PT and in light red for FOR005-GL. The data show the irreversible thermal unfolding of both V_L domains.

Figure S3

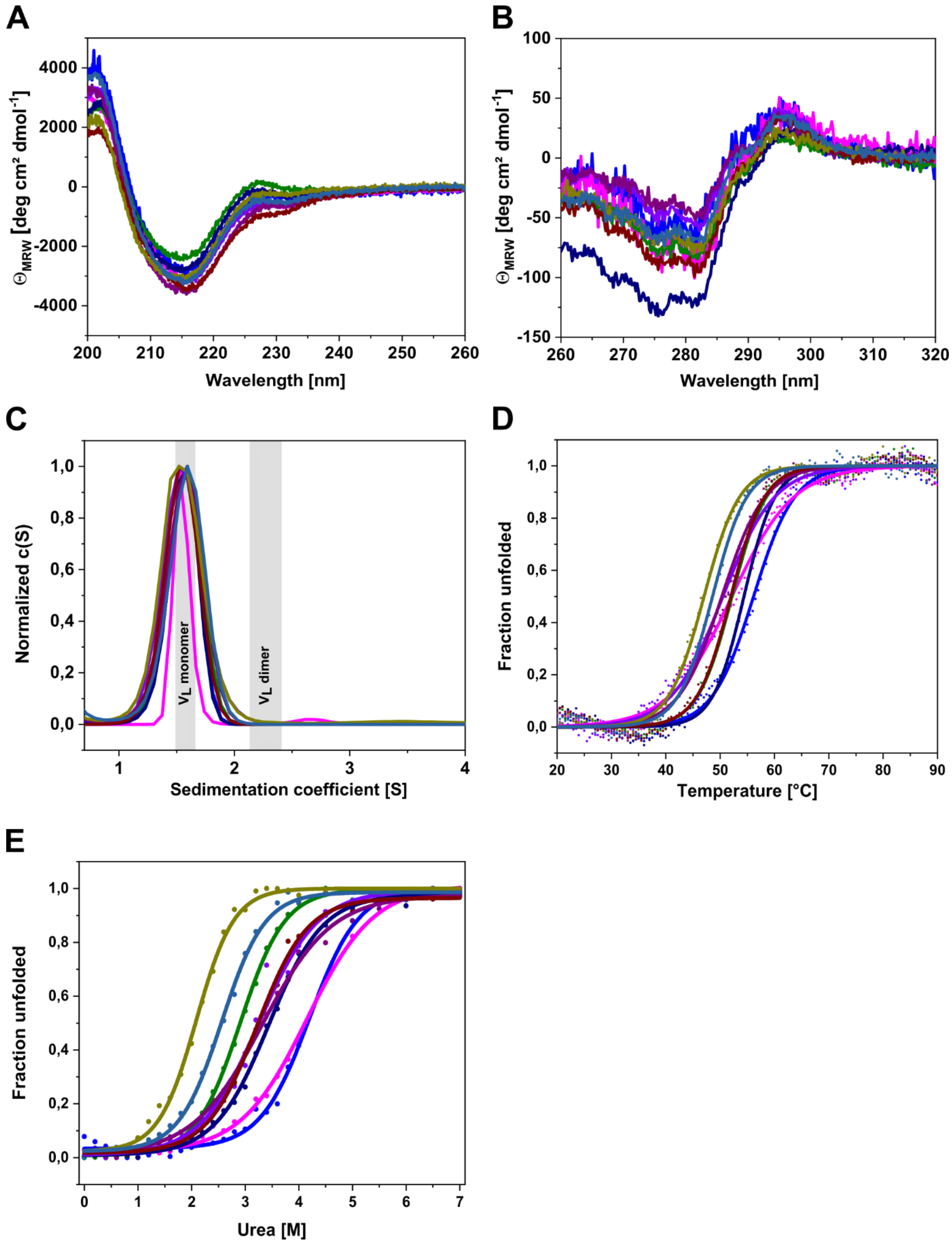
A
Θ_{MRW} [deg cm² dmol^{-1}]
4000
2000
0
-2000
-4000
200
210
220
230
240
250
260
Wavelength [nm]
B
Θ_{MRW} [deg cm² dmol^{-1}]
100
50
0
-50
-100
-150
260
270
280
290
300
310
320
Wavelength [nm]
C
Normalized c(S)
1,0
0,8
0,6
0,4
0,2
0,0
V_L monomer
V_L dimer
1
2
3
4
Sedimentation coefficient [S]
D
Fraction unfolded
1,0
0,8
0,6
0,4
0,2
0,0
20
30
40
50
60
70
80
90
Temperature [°C]
E
Fraction unfolded
1,0
0,8
0,6
0,4
0,2
0,0
0
1
2
3
4
5
6
7
Urea [M]

Figure S3: Biophysical characterization of FOR005-GL single and double mutants.

A) Far-UV CD spectra of FOR005-GL mutants Y31S (blue), Y48F (pink), G49R (green), N51S (dark blue), G94A (violet), Y31S/G94A (purple), Y48F/G94A (brown), G49R/G94A (dark yellow), and N51S/G94A (pale blue). All variants share the same secondary structure.

B) Near-UV CD spectra of FOR005-GL mutants (color code as in A) indicate a highly similar tertiary structure for all mutants.

C) Analytical ultracentrifugation of FOR005-GL single and double mutants shows that all proteins are monomeric in solution. Therefore, the mutations have no influence on the quaternary structure of the V_L domain. Grey bars indicate the approximate sedimentation regions of monomeric V_Ls (1.5 - 1.6 S) and V_L dimers (2.2 – 2.4 S), respectively.

D) Thermal transitions between 20 and 90 °C of FOR005-GL mutants recorded by CD at 205 nm. For transitions a heating rate of 1 °C/min was applied and 10 µM protein were used in a 1 mm quartz cuvette.

E) Urea induced unfolding transitions were recorded in triplicates at 25 °C in a Tecan Infinite M Nano+ plate reader using 1 µM protein and increasing concentrations of urea. Samples were equilibrated over night at room temperature, excitation wavelength was 280 nm and emission was measured from 300 – 400 nm. Fluorescence intensities of the triplicates were averaged, normalized, and fitted with Origin.

Figure S4

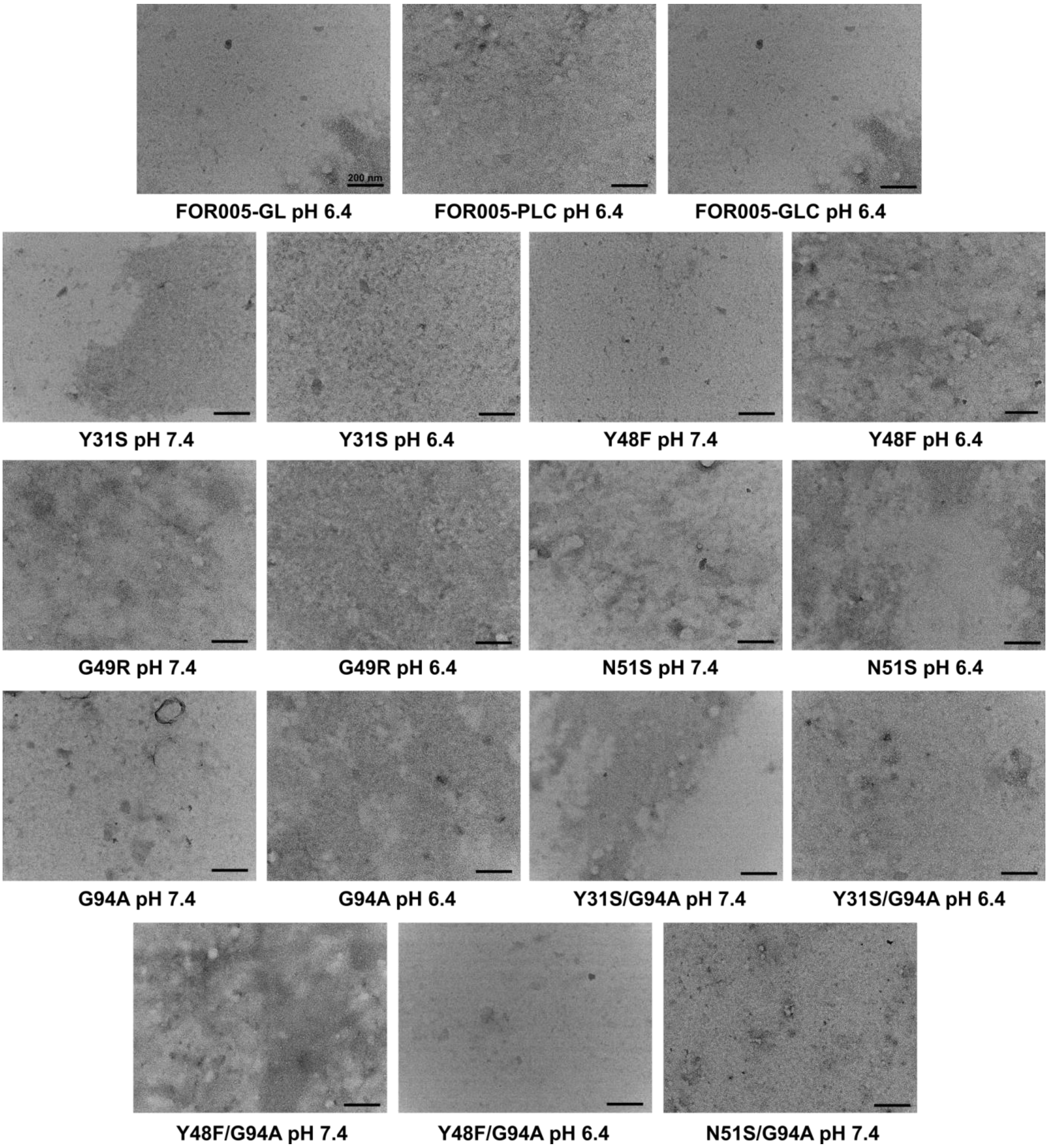

Figure S4: TEM micrographs of the remaining samples that did not exhibit a rise in ThT fluorescence. TEM grids were prepared by negative staining with uranyl acetate after ThT assays were completed. The scale bar represents 200 nm.

Figure S5

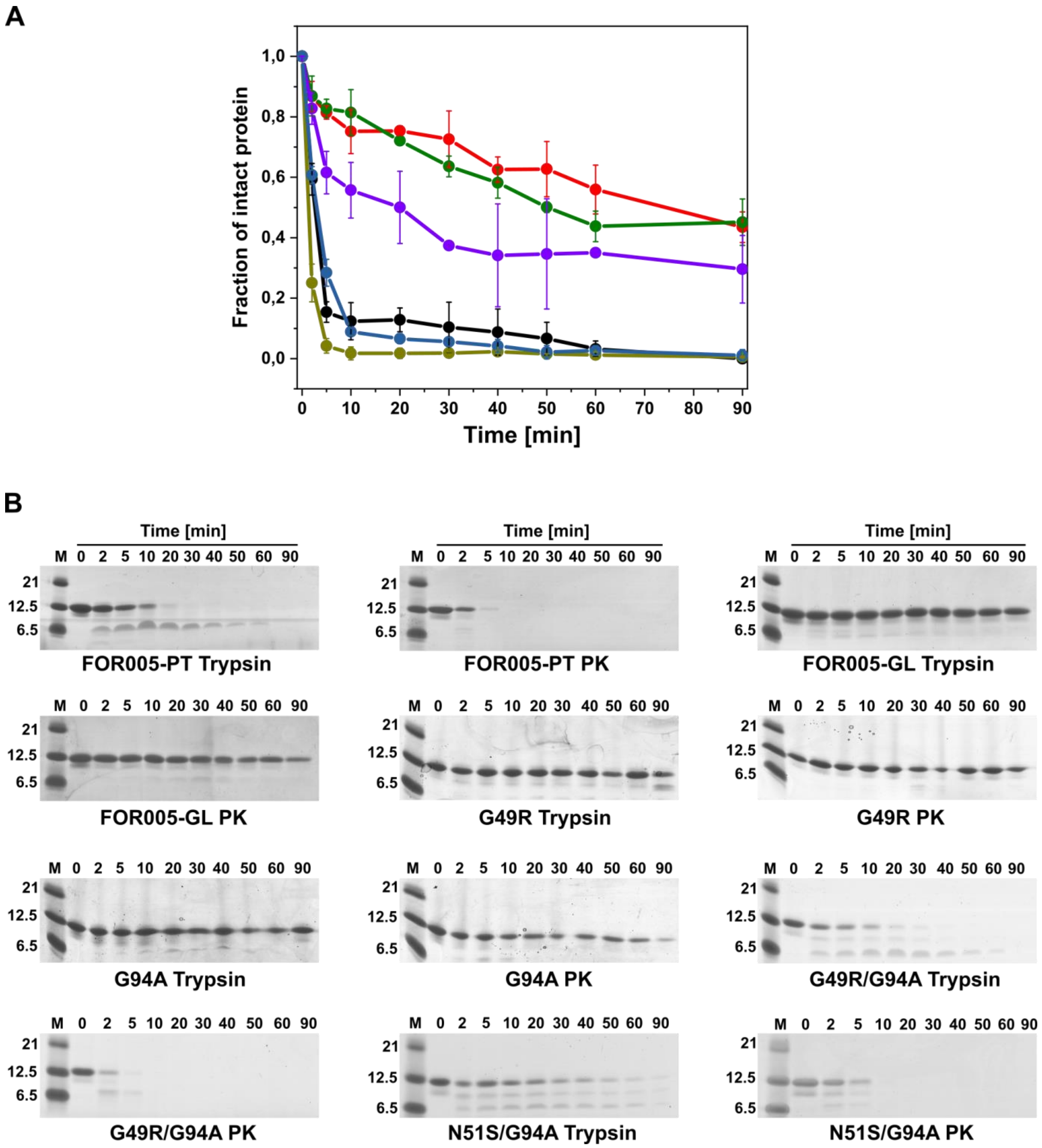

Figure S5: Limited Proteolysis of FOR005 constructs gives information about conformational dynamics.

A) Quantified limited proteolysis of FOR005-PT (black), GL (red), G49R (green), G94A (violet), G49R/G94A (dark yellow), and N51S/G94A (pale blue) using proteinase K (PK). A protein/protease ratio of 150/1 (w/w) was applied and samples were analyzed on SERVA Prime 4 - 20 % SDS gels. Quantification of gel bands was performed with NIH ImageJ.

B) SDS gels of limited proteolysis experiments. The experiments were performed in triplicates, however, only one gel per proteolysis reaction is shown. SERVA protein test mixture 6 was used as marker (M).

Figure S6

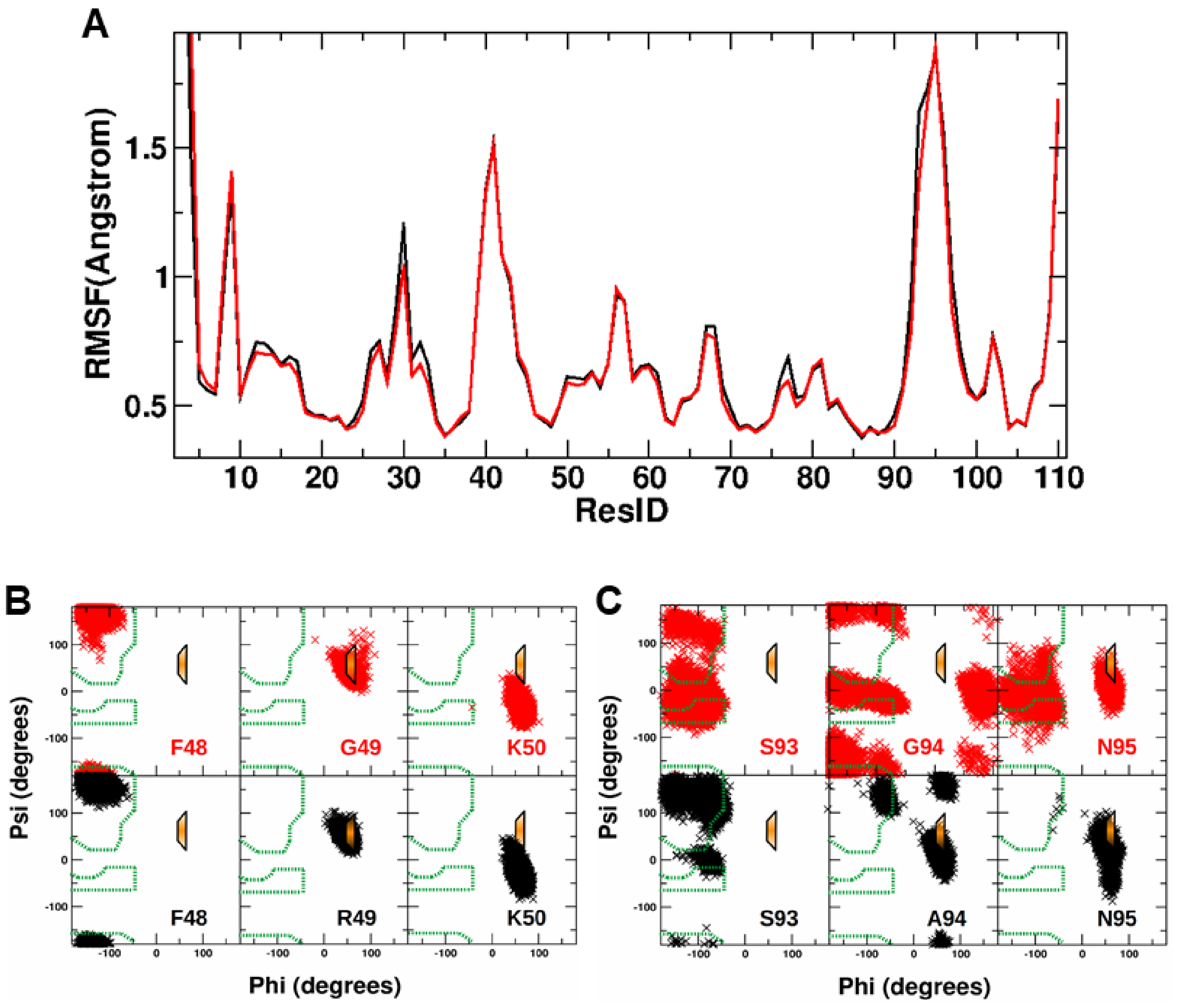

Figure S6: MD simulations show energetically unfavorable backbone conformations in CDR2 and CDR3 for FOR005-PT V_L and the R49G and A94G double substitution.

A) Root-mean-square fluctuations (RMSF) observed in MD simulations (1 µs, at 310 K) along the residue sequence for the FOR005-PT V_L variant (black line) and the R49G and A94G double substitution (red line).

B) Sampled backbone dihedral angles phi and psi plotted as Ramachandran plots for residues 48-50 observed during 1 µs simulations of FOR005-PT V_L (black dots) and the R49G and A94G variant (red dots). Favorable regions for non-glycine residues are indicated by a green dashed boundary in the Ramachandran plots and a regime favorable for glycine but less for other amino acids is indicated in orange with a blue boundary.

C) Same as B) but Ramachandran plots for residues 93 - 95 observed during 1 µs simulations of FOR005-PT V_L (black dots) and the R49G and A94G variant (red dots).

Figure S7

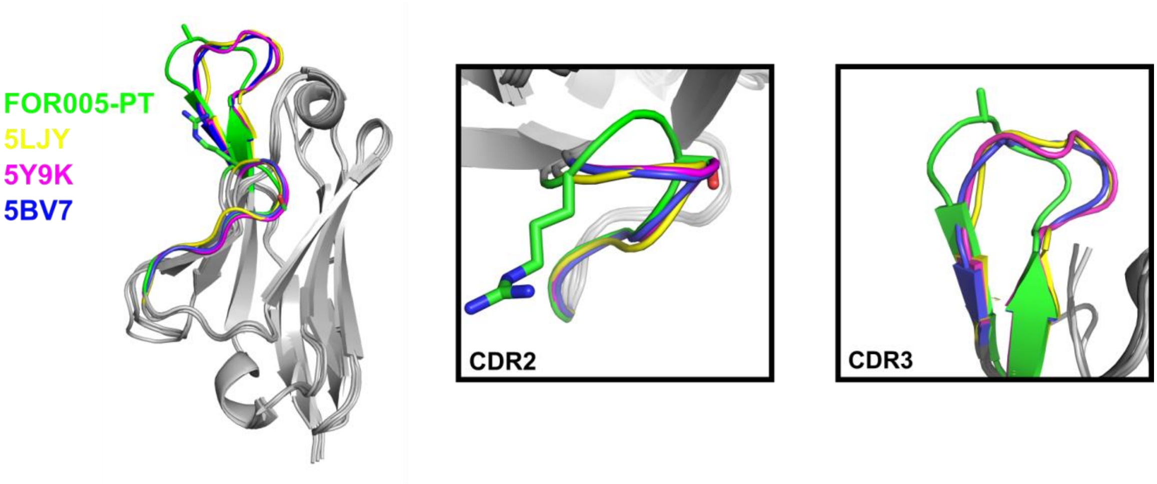

Figure S7: Structural alignment of FOR005-PT with three highly similar, non-amyloidogenic V_L domains shows the changed CDR loop conformation representing a deviation from canonical CDR classes. We searched PDB for primary sequences with high sequence similarity to FOR005-PT and aligned the crystal structures of the V_L monomers using PyMOL. The CDR2 and CDR3 regions are colored as follows: FOR005-PT = green, 5LJY = yellow, 5Y9K = magenta, 5BV7 = blue. The conformational differences between the CDR loops could provide a possible explanation for the observed destabilizing effects of CDR mutations.
